# Attitude, Perception, and Knowledge of COVID-19 Among General Public in Pakistan

**DOI:** 10.3389/fpubh.2020.602434

**Published:** 2020-12-09

**Authors:** Sammina Mahmood, Tariq Hussain, Faiq Mahmood, Mehmood Ahmad, Arfa Majeed, Bilal Mahmood Beg, Sadaf Areej

**Affiliations:** ^1^Department of Botany, Division of Science and Technology, University of Education, Lahore, Pakistan; ^2^College of Veterinary and Animal Sciences, Jhang, Pakistan; ^3^Lyallpur Business School, Government College University, Faisalabad, Pakistan; ^4^Department of Pharmacology, Riphah International University, Lahore, Pakistan; ^5^Department of Pharmacology and Toxicology, University of Veterinary and Animal Sciences, Lahore, Pakistan

**Keywords:** knowledge, perception, awareness, behavior, practices, public, COVID-19, Pakistan

## Abstract

The World Health Organization has acknowledged coronavirus disease 2019 (COVID-19) disease as a pandemic. Efforts are being made all over the world to raise awareness to prevent the spread of the disease. The goal of this study was to assess the attitude, perception, and knowledge of Pakistani people toward COVID-19 disease. We conducted a cross-sectional survey in which a questionnaire of 17 questions was transformed online on Google forms and was sent to random individuals online. A total of 1,000 questionnaires from individuals throughout Pakistan were evaluated. The results revealed that 42.9% of the participants knew about COVID-19 through social media, the largest source of information. Most of the participants (48.3%) started working from home amid the lockdown; 39.9% of the participants reported that they wash their hands every hour, and 56.9% participants are using a surgical mask. About thermal scanners, 30.5% of the people answered they may be effective, and 46.0% of the people think COVID-19 is a bioweapon; 59% of the participants think everyone is susceptible, whereas 83.9% of the people recognize fever as a primary symptom; 65.2% of the people are practicing social distancing, whereas 85.1% of the people think social gatherings causes spread of the disease. In general, participants had a good knowledge about the disease and a positive attitude toward protective measures. The effective measures are being taken by the government and the public; still, there remains a need for further awareness campaigns and knowledge of safe interventions to combat the spread of disease.

## Introduction

At the time when the whole world is fighting against the brisk irrepressible coronavirus disease 2019 (COVID-19), assessing the perception of a relevant population regarding necessary safety measures and their way of dealing with such a situation can help in better understanding of people's psychology. It will assist in better understanding of methodologies to counsel them in a way that leads to general public safety besides restraining the spread of the disease (1). Education programs regarding mental well-being via various communication platforms have been conducted vastly during the current breakout for ordinary people and health care professionals (2). By February 8, 2020, 29 books regarding COVID-19 were published, of which 11 were related to the mental well-being of people including a book for counseling and self-safety against COVID-19 publicized by the Chinese Association for Mental Health (3).

Household surveys based on the compact segment or grid-based geographical information system sampling usually take weeks to months to complete (4). Phone-based surveys have widely been conducted since 1980, but people's communication methods have changed a lot since then; for example, Gallup Poll Social Series received only a 7% response rate via phone in 2017 (5). Keeping these limitations in mind, rapid online surveys are reasonably easy to conduct and get completed fast compared to other conventional survey methods. The online survey also demands minimum human resources, besides those needed for preparing a questionnaire, to reach a vast range of respondents in a minimum period and also allow continuous survey monitoring (6). Thus, it can be considered as a powerful tool to collect information in such a global outbreak situation.

The COVID-19 that was declared a pandemic by the World Health Organization (WHO) on March 13, 2020, after the widespread of the disease in Europe, and the drastic number of deaths in Italy is exceptionally transmissible (7). The current scenario has shown quite a noticeable impact on people's mental well-being besides their physical well-being. It has changed their perception about life, and their priorities regarding daily life routine have also been affected (8). Such a global situation can only be controlled with people's consent to behave in a particular manner instructed by health care providers such as frequent hand washing, using the facemask, avoiding gatherings, and maintaining permissible distance (9).

Another factor that needs to be tackled is misconceptions related to the disease besides the disease itself—keeping into consideration the given fact, WHO has designed a particular page named “myth buster” to tackle such misconceptions, which are even more harmful than the disease itself. Such outbreaks have always been accompanied by a tsunami of misinformation and misuse of drug therapies (10). However, in an era of fast communication technologies, such misconceptions get amplified and spread at a pace faster than the real facts through social media. Such fake pieces of information and concepts could be drastically damaging for the general public who blindly follow any information they get to run for safety (11).

In a study conducted in China, most people (60.81%) who spent 20–24 h at home did not show signs of the disease, and very few contacted any patients with COVID-19 history. Internet was the primary source of health-related news updates for these people. Almost 90% public asked for regular information update regarding ways of disease transmission, preparation on new medications for the disease, precautionary measures to be kept in focus while traveling, ways other countries are handling the pandemic, areas more affected by the virus, and related details. Almost 70% population was appeased with details regarding health care provided to them. More than half of the population used to wash their hands regularly with soap and used facemasks as a precaution regardless of assessing the presence of the disease symptoms in them (12).

This rapid survey was designed to determine knowledge, attitude, and perceptions of COVID-19 among educated general public in Pakistan. The in-depth analysis was carried out through online assessment to better understand the knowledge and risk perception about COVID-19 outbreak in Pakistan.

## Methodology

A questionnaire was designed, keeping into consideration the prevalent attitudes and beliefs of the general public of COVID-19. Some past guidelines were kept into consideration while designing the questionnaire (13). The form was first assessed by a few healthcare professionals from various domains. The corrections and adjustments were made as per their suggestions. The grammatical errors were corrected using Grammarly by adjusting the goals of questions according to audience, formality, domain, tone, and intent. The audience and domain were selected as “general,” whereas the formality, tone, and intent were adjusted as “neutral,” “analytical,” and “describe,” respectively. The form was then distributed among 20 participants, and their feedback was taken. This was to evaluate how each participant perceives the question. Their feedback, if any, was also taken into consideration to make any change to the questionnaire.

The questionnaire was then transformed online on Google forms and was sent to random individuals online (e-mail, WhatsApp, and Facebook groups). A total of 2,000 individuals were invited to fill the questionnaire throughout Pakistan, including Azad Jammu and Kashmir. The e-mail address was a mandatory field to contact an individual in case of any confusion. The header of the form reads the consent policy, and the objectives of the study were made clear. The participation was completely voluntary. The participants may leave the form any time without submitting the form. The form was kept open for 10 days, and no responses were accepted after it. The first question requires the participant to disclose their English fluency. Since English has been the official language of the country, it is not widely used and understood by the general public. Anyone who responded non-fluency in the English language was eliminated from the study.

Similarly, the second question asked the individuals whether they heard of COVID-19 or 2019-nCoV or coronavirus disease in 2019. Those who answered “no” to this question were also excluded from the study. All other responses were accepted. All the questions in the form were mandatory except for the name of the participant.

### Statistical Analysis

The results were exported from Google forms and saved in CSV format. MS Excel 2013 and SPSSv21 were used to evaluate data. The results were expressed as mean, median, interquartile ranges (IQRs), and percentages. A χ^2^ test was applied to assess any correlation between categorical variables.

## Results

A total of 1,159 responses were received, making a response rate of 57.95% response rate. Of them, 30 participants (2.59%) disclosed that they do not have fluent English and were eliminated. One hundred nine participants (9.41%) had never heard of COVID-19. Twenty (1.73%) responded that they do not have fluent English, and they had never heard of the disease. These individuals were also excluded from the study. Hence, only 1,000 responses were evaluated throughout the study. Of these, 62.1% (621) of the sample population were males, and 37.9% (379) were females. The mean ± standard deviation and median (IQR) age of the participants were 25.39 ± 6.07 years and 24.0 (21.0–29.0) years, respectively.

### Demographic Details of the Respondents

**Figure 1:**
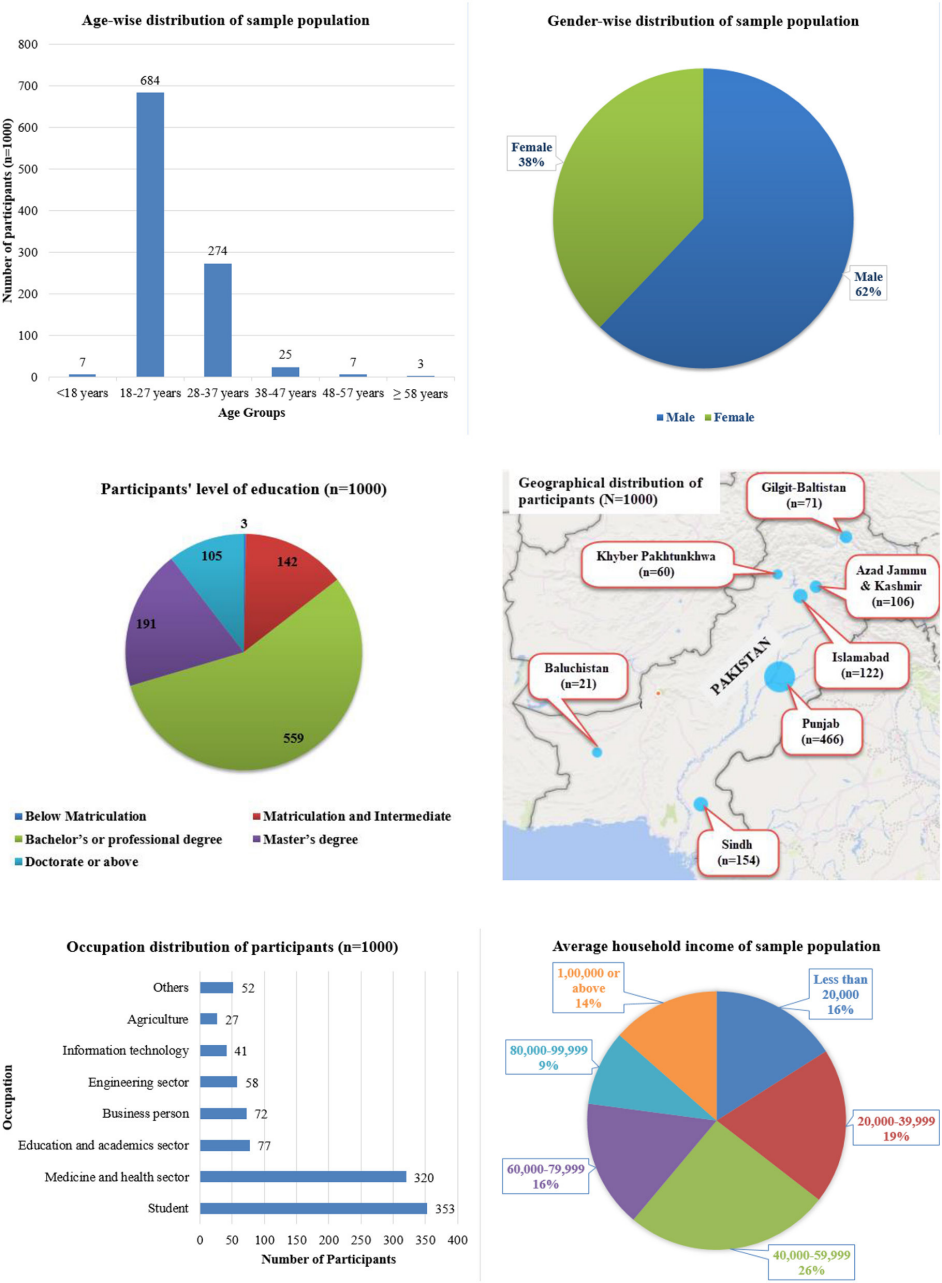
Demographical features of the participants.

Figure 1 given below show the demographic characteristics, i.e., age, gender, area of residence, education, occupation, and total household income of the respondents who filled the online survey: age: the maximum number of people were between the age of 18–27 years (68.4%); gender: 62.1% of males and 37.9% of females participated in the study; area of residence: most people who participated in the study were from Punjab (46.6%) and minimum from Baluchistan (2.1%); education: a majority of the population (55.9%) was bachelor or professional degree holders, followed by master's degree holders (19.1%) and a minimum number of people were below the matriculation level (0.3%); occupation: mostly, students (35.3%) and professionals from the health sector (32.0%) filled the online survey; and total household income: the majority of people (25.6%) who participated in the study have a household income between 40,000 and 59,999 PKR.

### Attitudes, Behavior, and Perceptions About COVID-19

The behavior and attitude of people toward the COVID-19 pandemic play an essential role in its spread, and according to our survey, more than three-fourths of the respondents knew about through social media (42.9%) and electronic media (41%). A small number of people had direct exposure (6.2%), and the least people came to know about it through print media (1.2%). The people who had jobs were inquired about their work handling, and nearly half of the respondents (48.3%) were working from home. Followed by that, there was a complete shutdown of work for those who had businesses (21.7%), and a small number of respondents continued their job as before (20.1%).

When asked about their social distance practices (65.6%), people responded that they have not been to any gatherings at all, while 3.6% responded they have been to gatherings many times. Upon inquiring about their hygiene habits and hand-washing frequency as directed by the health care professionals, the majority of people responded that they washed their hands after every hour (39.9%), followed by those who washed them few times a day (35.3%), and 1.6% said that they do not wash their hands.

Using a mask during the COVID-19 outbreak has been advised by the health care professionals, and upon asking which mask did the participants of our study use as a preventive measure, more than half of them (56.9%) used the surgical mask, followed by cloth mask (18.0%) and N95 mask (6.3%). People who did not use masks were 17.8%, and 1% of the participants did not know about it. The perception of people toward thermal scanners showed that a small percentage of people (24.8%) considered them useful, whereas 30.1% thought it is not an effective way of scanning for the virus.

There has been much debate about COVID-19 being a bioweapon or not, so we asked this from the participants of our study, and 46% think it is a bioweapon, and 42.6% think it is not. Only 11.4% responded “maybe” toward this question. The responses are recorded in Table 1.

**Table 1:** Attitudes, behavior and perceptions about COVID-19.

	***n (%)***
**How did you come to know about COVID-19?**	
Social Media	429 (42.9%)
Electronic Media (TV/Radio)	410 (41.0%)
By Family or Friends	87 (8.7%)
Direct Exposure	62 (6.2%)
Print Media (Newspaper/Articles)	12 (1.2%)
**How are you handling work during this outbreak?**	
Working from home	483 (48.3%)
Business is completely shut down	217 (21.7%)
Continuing job as before	201 (20.1%)
Left job for my safety	99 (9.9%)
**Have you been to gatherings since then?**	
Many times	36 (3.6%)
A few times	212 (21.2%)
Once	96 (9.6%)
Not at all	656 (65.6%)
**How often do you wash your hands as stated by health professionals?**	
After every half hour	232 (23.2%)
After every hour	399 (39.9%)
Few times a day	353 (35.3%)
I don't wash them	16 (1.6%)
**What type of mask do you use?**	
Surgical Mask	569 (56.9%)
Cloth mask	180 (18.0%)
N95 mask	63 (6.3%)
I do not use a mask	178 (17.8%)
I don't know	10 (1.0%)
**Are thermal scanners effective in detecting patients with corona-virus?**	
Yes	248 (24.8%)
May be	305 (30.5%)
Only if patient has fever	215 (21.5%)
No, it is not 100% effective way of detection	231 (23.1%)
**Do you think COVID-19 is a bioweapon?**	
Yes	460 (46.0%)
No	426 (42.6%)
May be	114 (11.4%)

### Knowledge of COVID-19 Among Participants

The participants had sensibly well knowledge about the diseases, and 83.9% recognized fever, 80.8% recognized cough, and 68.6% recognized shortness of breath as the three main symptoms of COVID-19. Upon asking, 59.0% responded that everyone is susceptible to this disease. About two-thirds of participants responded that the most effective way of controlling the disease is social distancing (65.2%) and frequent hand washing (63.8%). More than half of the respondents (54.4%) stated that using masks as directed by health care professionals is effective in disease control, and approximately 45.0% responded that avoiding sick people is also an effective preventive measure toward COVID-19. Overall dietary habits and lifestyle were also inquired. The response from 35.6% was that eating fruits with vitamin C is an effective in the prevention of COVID-19, followed by taking steam (responded by 23.5%), rinsing the nose and doing gargles repeatedly (23.2%), taking honey, black cumin, and garlic (16.8%), taking green tea (14.8%), exposure to the sun (8.4%), pneumonia vaccine shot by 3.4%, and taking antibiotics was responded by 1.9% of the participants.

We asked the common causes of transmission of the disease, and 85.1% responded to social gatherings, and 69.2% of the participants thought that the handshakes are the main culprits in the spread of the disease. Handling banknotes and currency were also responded as a positive mean by about half of the participants (46.4%). In comparison (40.4%), people answered that not maintaining a distance of at least 6 feet can cause transmission of the disease. Other frequently touched items can also be the cause of spreading the diseases, and upon asking, 35.2% thought doorknobs, 22.8% through mobile phones, 15.0% thought used gloves and masks, and 6.8% thought clothes are a mode of transmission of COVID-19 virus among the population. The responses are recorded in Table 2.

**Table 2:** Knowledge of COVID-19 among participants (*n* = 1,000).

**Who is more likely to get infected with coronavirus?**	
Everyone is susceptible	590 (59.0%)
Old age	443 (44.3%)
Infants and Children	204 (20.4%)
Adults	30 (3.0%)
I don't know	14 (1.4%)
**Symptoms indicating that patient is infected with coronavirus?**	
Fever	839 (83.9%)
Cough	808 (80.8%)
Shortness of breath	686 (68.6%)
Flu	436 (43.6%)
Body pain	227 (22.7%)
No symptoms	203 (20.3%)
Stomach problem	32 (3.2%)
Skin rash	23 (2.3%)
Nose bleed	18 (1.8%)
**Effective measures for controlling COVID-19?**	
Social distancing	652 (65.2%)
Frequent hand washing	638 (63.8%)
Using masks and gloves	544 (54.4%)
Avoid contacting sick people	450 (45.0%)
Avoid touching face	392 (39.2%)
Eating fruits with Vitamin-C	356 (35.6%)
Taking steam	235 (23.5%)
Rinsing nose and doing gargles repeatedly	232 (23.2%)
Taking honey, black cumin, and garlic	168 (16.8%)
Taking green tea	148 (14.8%)
Exposure to sun	84 (8.4%)
Pneumonia vaccine shot	34 (3.4%)
Taking antibiotics	19 (1.9%)
**Most common causes of coronavirus transmission?**	
Social gatherings	851 (85.1%)
Handshakes	692 (69.2%)
Money or bank notes handling	464 (46.4%)
Standing at 6 feet distance	404 (40.4%)
Door knob and handles	352 (35.2%)
Mobile phones	228 (22.8%)
Used gloves and masks	150 (15.0%)
Clothes	68 (6.8%)

### Relationship Between Participants' Demographic Characteristics and Their Responses

Associations of different characteristics were made against the participants' knowledge of COVID-19. First, education levels of the participants were evaluated against the use of mask by the participants. It was observed that the use of make was significantly less among individuals with lower education levels as compared to individuals with higher education (*P* < 0.05). However, non-significant relationships were present between education level and other aspects of our study such as attending social gatherings, frequent hand washing, and COVID-19 as a bioweapon. Second, income levels of the participants were analyzed against various factors. A significant relationship was found among the income level of the participant and their affinity to join social gatherings during the COVID-19 pandemic. It was observed that respondents with lower income levels were more likely to attend gatherings as compared to higher-income respondents. Similarly, income level was also found to be associated with the subjects' use of mask. Participants with higher income levels were wearing N95 and surgical masks, whereas cloth masks were usually used by lower-income respondents. Terminally higher- and lower- income category subjects wore fewer masks as compared to middle-income-range groups. More participants in the lower-income category had to leave their jobs or had their business shut down during the COVID-19 pandemic as compared to higher-income respondents. Likewise, more women have left their jobs as compared to their male counterparts. However, business shutdown trend mainly affected the male gender. Male respondents were gathering more frequently as compared to the females (*P* < 0.05). Moreover, the males were found to wear mask more often than the females. Females usually wore N95 masks, whereas male respondents relied more on surgical masks or cloth masks. On the contrary, no significant associations were present among all the characteristics with hand-washing practices and the use of COVID-19 as bioweapons (Table 3).

**Table 3:** Statistical Significance between participants' demographic characteristics and their responses.

**Sr. No**.	**Demographic characteristics**	**Attitudes of respondents**	***p*-Value**
1	Education level	Use of mask	<0.05
		COVID-19 as bioweapon, gatherings, hand washing	>0.05
2	Income level	Gatherings, type of mask, work status	<0.05
		Hand washing, COVID-19 as bioweapon	>0.05
3	Gender	Work status, gatherings, use of mask	<0.05
		Hand washing, COVID-19 as bioweapon	>0.05

*Pearson Chi-square test was applied and values were significant (p < 0.05)*.

## Discussion

The in-depth analysis of perceptions and knowledge of COVID-19 was carried out through online assessment among the general public of Pakistan. It took only 10 days to obtain 1,159 responses of knowledge and a perception-based questionnaire containing 17 questions, of which responses of 1,000 adults were analyzed. It appears from the results that most of the participants came to know about the disease COVID-19 or SARS-CoV-2 through social and electronic media. Although these platforms are a natural source of access to the information and data around the world, it is still not the most reliable option to choose from. Fake news is often associated with such platforms as Facebook. For example, there had been much misinformation about the hydroxychloroquine to be used as a potential treatment for the COVID-19, which caused a shortage of that drug for those who actually needed them (14). The demographics of the participants, like age, sex, and ethnicity, indicated that most active participation was seen from male adults of Punjab province. The distribution of participants among education and household income showed that people came from mediocre but educated families. It might be due to the reason that Punjab is concentrated in population and has the highest literacy rate among other provinces (15); therefore, most people were able to respond to an online assessment survey form. The highest number of responses indicates the quality of the study was obtained by students mostly holding a professional or master's degree and health or medical professionals. This means that they carefully evaluated and answered the questions based on pertinent information. A similar trend was observed in a survey-based study among students of China (16).

Regarding the survey findings, the participants are observing social distancing and hand washing quite frequently. The general public appeared to have a compliant attitude toward the precautions to be taken for COVID-19 (17). The participants all over the region believe that typical surgical masks are most effective in protecting them from the COVID infection, and they overestimated the protective ability of a cloth mask over an N95 mask. In a similar study performed in Egypt, the general public seemed to believe that wearing a mask will save them for virus infection, but they do not know which mask is active (18). The WHO has not recommended that healthy people should wear masks; however, if someone is experiencing symptoms of the respiratory disease, which could possibly be a SARS-CoV-2 infection, they must wear an N95 mask for protective purposes (19). Thermal scanners are also being used at the grocery stores and banks, which are open in Pakistan during the pandemic, and a very few people responded that it might be an effective mean of scanning for COVID-19–positive patients. Likely as a result of this perception, it looks like even the educated people do not put their faith in the effectiveness of thermal scanners. However, it has been reported in the literature that thermal scanners have been used as an effective strategy at hospitals (20) and airports (21) for the screening of SARS-CoV-2 infection, and they have proven helpful.

There is a controversy about COVID-19 plague being a bioweapon, and according to our survey, almost half of the participants responded “yes,” and half of them responded “no,” whereas a small percentage responded, “maybe.” The results interestingly show that the perception of people equally supports both notions, i.e., COVID-19 being a natural pandemic or a possible bioweapon. The results are comparable to a study where the researchers explain how this infection has virtually created a downfall to the superpowers of the world by putting the entire world in quarantine and devastating the global economy (22).

In our collected responses, majority participants thought that everyone is susceptible to the novel coronavirus disease, while nearly the same number of participants responded that older people are more likely to get it. The results are similar to a Chinese study, which provides information about the COVID-19-affected people (23). The COVID-19 infection may be symptomatic or asymptomatic in many people; however, according to the result of our survey, the participants have recognized fever, cough, and shortness of breath as three primary symptoms of the infected person. The knowledge of our participants is in line with those presented by other epidemiological studies (23, 24).

The most crucial thing in a contagious disease is to be cautious of its mode of transmission and valid measures of its prevention. The participants in our survey have largely responded that social gatherings and intimacy such as handshakes are the leading cause of the spread of the SARS-CoV-2 infection, and the key to preventing and containing it is also in practicing social distancing and frequent hand washing. These responses show that the knowledge of the participants is up-to-date and in accordance with the guidelines of WHO (25, 26). We can relate the knowledge and perception of Pakistani people to Chinese containment of the COVID-19. They practiced control measures such as limiting social gatherings by shutting down cities and limiting the traffic throughout their country. They had confidence in winning the battle against coronavirus, so they used these measures wisely and were able to control the disease effectively with a low mortality rate (23).

The COVID-19 pandemic causes a huge global health crisis and impacts on large-scale behavior and attitude changes of the public (1). Proportion of asymptomatic patients in case of COVID-19 is high and an important feature of this disease. It is estimated that approximately 60% of all infections with mild symptoms or asymptotic cases might pass the virus to others (27). With the increasing number of COVID-19 cases globally, different countries including Pakistan have adopted precautionary measures, i.e., social distancing, frequent hand washing, and wearing mask to prevent its spread. Wearing facemask is an effective physical intervention against disease transmission (28). The use of facemasks has become extensive in developed and underdeveloped countries including Asia. Most of the people are using simple surgical mask to reduce the risk of getting COVID-19 infection (29). Public perception of health risk plays a vital role in the adoption of government measures to prevent spread of COVID-19, and these measures and actions had direct influence on lifestyles and attitude of people (30).

### Limitations and Strengths

This study is among a few studies that were conducted to evaluate the attitude, perception, and knowledge of general public regarding COVID-19. A huge number of participants took part in this study. As the study was conducted during lockdown, an online questionnaire was used for assessment. The study was limited to only those individuals who were able to read and write English language. Another limitation of this study was that most of the responses we received were from educated section of society who have access to the internet, and the rest of the public was hence excluded. A large number of respondents in this study were linked to various healthcare professions; therefore, this study may also have exhibited good knowledge of disease among participants. Unfortunately, the study may not have exactly represented all the proportions of our current society; however, it may reflect a general overview of the behaviors present in the society.

Because of limited representation of the participants, more studies like this are suggested to investigate other areas related to COVID-19 in Pakistan such as the economic burden and availability of the SARS-CoV-2 vaccines in the residents of low socioeconomic status. Our study was limited to the people who understand the English language. Furthermore, most of the respondents have a medical background, so the knowledge and behavior of these individuals affect the overall study results.

However, some recent studies have shown similar results as those of our study. In one study, it was shown that there is a high-level knowledge regarding the disease among the participants. But some myths are also prevalent among the public (31, 32). Such gaps need to be addressed in the education and awareness programs for better response toward COVID-19 (33).

## Conclusion

In summary, the findings of our survey suggest that the Pakistani residents of a relatively mediocre socioeconomic status, particularly men and students with a medical background, have appropriate practices and optimistic approaches due to the peak of a COVID-19 infection period. Different opinion lies among each gender regarding the perception of COVID-19. The education and health programs have aimed at improving the knowledge of the general population about COVID-19, and they are maintaining safe practices and optimism in people's attitudes.

## Data Availability Statement

The original contributions presented in the study are included in the article/supplementary materials, further inquiries can be directed to the corresponding author/s.

## Ethics Statement

Ethical review and approval was not required for the study on human participants in accordance with the local legislation and institutional requirements. The patients/participants provided their written informed consent to participate in this study. Written informed consent was obtained from the individual(s) for the publication of any potentially identifiable images or data included in this article.

## Author Contributions

SM and FM conceived and written the manuscript. TH did final review and approved the manuscript. MA did statistical analysis and manuscript writing. AM designed the manuscript and did data collection. BB and SA compiled results and editing of manuscript. All authors contributed to the article and approved the submitted version.

## Conflict of Interest

The authors declare that the research was conducted in the absence of any commercial or financial relationships that could be construed as a potential conflict of interest.
